# Iron encapsulated in single-walled carbon nanotubes for obtaining the evidence of improved coulombic efficiency and improving the lithium battery performance of ZnO anodes†

**DOI:** 10.1039/c8ra00480c

**Published:** 2018-03-23

**Authors:** Jiaxin Li, Mingzhong Zou, Weijian Huang, Chuxin Wu, Yi Zhao, Lunhui Guan, Zhigao Huang

**Affiliations:** CAS Key Laboratory of Design and Assembly of Functional Nanostructures, Fujian Institute of Research on the Structure of Matter, Chinese Academy of Sciences Fuzhou Fujian 350002 P. R. China; Fujian Provincial Key Laboratory of Nanomaterials, Fujian Institute of Research on the Structure of Matter, Chinese Academy of Sciences Fuzhou Fujian 350002 P. R. China guanlh@fjirsm.ac.cn +86 591 83792835 +86 591 83792835; College of Physics and Energy, Fujian Normal University, Fujian Provincial Key Laboratory of Quantum Manipulation and New Energy Materials Fuzhou 350117 China zghuang@fjnu.edu.cn +86 591 22867577 +86 591 22867577; Fujian Provincial Collaborative Innovation Center for Optoelectronic Semiconductors and Efficient Devices Xiamen 361005 China

## Abstract

Cycling coulombic efficiency including the 1^st^ cycle is a crucial factor for nano-carbon based anodes. How to improve their coulombic efficiency and further prove whether the additional reversible capacity produced from the SEI film in the 1^st^ cycle is an obstacle for their possible commercial application in Li ion batteries (LIBs). For this aim, a novel composite of Fe-encapsulated single-walled carbon nanotubes (Fe@SWNTs) with special nano-structure was designed and used as an anode material for LIBs. The resulting Fe@SWNT anode can provide much larger coulombic efficiency of 53.1% in the 1^st^ cycle than 35.6% for pure SWNTs, implying the value increment reached ∼50%. The Fe@SWNTs can exhibit an reversible capacity of 420 mA h g^−1^ after 300 cycles and excellent rate performance at room temperature, being obviously better than 275 mA h g^−1^ for a SWNT anode. The origination of this extra improved reversible capacity can be confirmed to be derived from the reversible reaction of SEI film activated by the Fe catalyst. Meanwhile, the Fe@SWNT anodes exhibited superior low-temperature (at 5 and −15 °C) electrochemical performance, which should be associated with an improved effect of the highly conducting Fe at low temperature, and with the activation of catalyst Fe on the reversible capacity. In addition, when Fe@SWNTs were developed as carriers for attaching ZnO, the ZnO/Fe@SWNTs can deliver much better LIB performance than anodes of pure ZnO and ZnO/SWNTs. Thus, catalyst modification supplied a promising route to obtain improved coulombic efficiency and reversible capacity for LIB nano-carbon based anodes.

## Abstract 2

Fe filler enables catalytic activation for the anode to obtain improved lithium battery performance.

## Graphical abstract

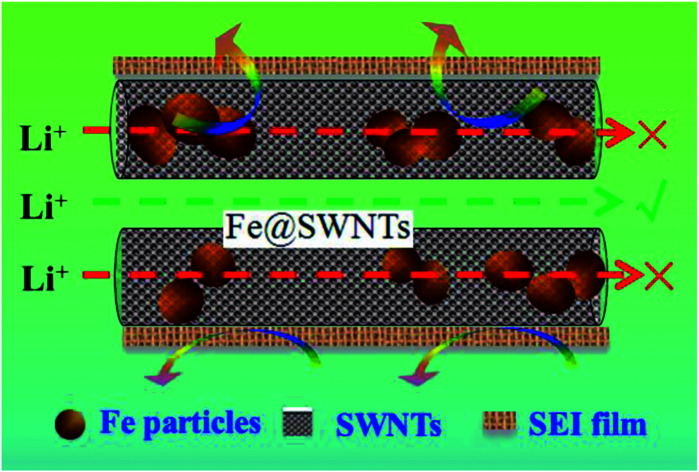

## 1. Introduction

Nowadays, lithium ion batteries (LIBs) are under intensive research and development, as they are characterized by promising theoretical gravimetric and volumetric energy.^1,2^ Though commercial graphite has been developed with improved performance, its power density is still too low to meet the increasingly growing demand for high-level applications for LIBs.^2,3^ As expected, anodes of nano-materials, especially for nano-carbon materials with good conductivity, beneficial for Li^+^ transfer, have been believed to be promising anode materials for LIBs.^4,5^ However, the irreversible capacity loss in the 1^st^ discharge, the poor coulombic efficiency and the relatively low capacity for anodic nano-carbon materials hampers their high-level applications in LIBs.^5,6^ It is of vital importance to effectively overcome these issues for anodic nano-carbon materials.

Recently, several useful strategies have been proposed to overcome this obstacle, including the utilization of conductive metal nanoparticles covered on the materials and the utilization of metal catalysts attached on the surface of nano-carbon anode materials.^7–18^ As a typical example, Zhou's group reported that anode of carbon materials loaded with nano-sized metal catalyst can reduce some solid electrolyte interphase (SEI) components and further improve their reversible capacities.^9,10^ They certified that anodes of Ni nanoparticles (NPs) highly dispersed in N-containing carbon nanosheets can afford an unexpected reversible capacity of 1051 mA h g^−1^ after 30 cycles and 635 mA h g^−1^ after 100 cycles at the current density of 200 mA g^−1^.^9^ Subsequently, iron carbide (Fe_3_C) has attracted interests of researchers in LIBs due to its high catalytic activity, sufficient thermal stability and extreme hardness.^10,11^ Some recent reports also revealed that nano-carbon anodes containing composites of Fe_3_C can reduce some SEI components and further improve the reversible capacities for LIBs.^12–16^ Our group previously reported that the novel composites of Fe/Fe_3_C–CNFs can exhibit a high reversible capacity at room temperature, and deliver a high capacity of 250 mA h g^−1^ at 400 mA g^−1^ even after 55 cycles at a low temperature of −15 °C.^17^ The superior electrochemical performance of the Fe/Fe_3_C–CNF anodes is associated with the synergistic effect of the catalytic effect of Fe_3_C and the highly conducting Fe at low temperature. Especially, it is recently reported that Fe_3_C possessing good catalytic activity can prompt anodes of Fe_3_O_4_@Fe_3_C–C yolk–shell nanospindles to obtain excellent LIB performance.^18^ It is believed that the present Fe_3_O_4_@Fe_3_C–C yolk–shell nanospindles are the most efficient Fe_3_O_4_-based anode materials ever reported for LIBs. Subsequently, an important question comes around, how to prove whether the additional capacity derived from the reversible reaction of SEI film is desired for nano-material anodes.

It is generally recognized that single-walled carbon nanotubes (SWNTs) with unique structure have attracted considerable interest of scientists.^19–23^ In particular, SWNTs have been proven to be a good nano-vessel for investigating confined reaction.^19–23^ Besides, confined iron NPs have become of great interest in catalytic energy conversion and storage due to its thermal stability and catalytic activity.^24,25^ Based on the above discussion, the remarkable characteristics of a nano-vessel structure for SWNTs and the confined iron NPs motivated us to find out whether we can rationally design an encapsulated structure of Fe-based nanomaterials with sufficient catalytic activity for LIBs, and further provide evidence for proving the above mentioned hypothesis.

In this paper, we attempt to introduce nano-sized iron particles in SWNTs *via* a simple vapor phase method coupled with heat treatment and form a special hybrid material of Fe@SWNTs, with the aim of proving its electrochemical mechanism for LIBs. With the iron filling, Fe@SWNT anodes exhibited obviously better LIB performance including low-temperature performance compared with the pure SWNTs. Importantly, the result proves that the additional capacity produce from the reversible reaction of SEI film. In addition, we preliminarily used them as high performance carriers for ZnO NPs, forming ZnO/Fe@SWNT anodes. The reversible capacity of ZnO/Fe@SWNTs (containing 65 wt% ZnO) remains ∼575 mA h g^−1^ measured at 600 mA g^−1^ after 150 cycles, much better than those of ZnO NPs attached on carrier of pure SWNTs.

## 2. Experimental part

### 2.1. Materials synthesis

In this work, all chemicals were of analytical grade and used as received. The details of preparation for SWNTs and ferrocene@SWNTs (Fc@SWNTs) were presented in our earlier work.^19–21^ A single process was adopted to prepare Fe@SWNTs. The as-prepared Fc@SWNTs were annealed in a Ar flow at 500 °C for 1 h with a heating rate of 5 °C min^−1^ for forming Fe@SWNT composite. Fe particles outside of the SWNTs were removed by through washing in acid solution. The weight of Fe@SWNTs after washing is about 1.25 times greater than that of the empty SWNTs after encapsulation of Fe. Herein, the additional illustration for electrodes used for Raman test was presented in the ESI.†

The typical preparation of ZnO/SWNT or ZnO/Fe@SWNT composites is given as follows. The SWNTs or Fe@SWNTs were treated with concentrated HNO_3_ (16 mol L^−1^) in 140 °C for 14 h. The composites were prepared using Zn(CH_3_COO)_2_·2H_2_O and KOH as the starting materials. 40 mg Zn(CH_3_COO)_2_·2H_2_O was dissolved in 50 mL anhydrous ethanol, following which 56 mg KOH was dispersed in the as-prepared solution by ultrasonification for 15 min in ice-water bath. A stable and optically transparent ZnO solution was consequently obtained and further stirred for 12 h. And then, 50 mg Fe@SWNTs of 40 mg SWNTs were respectively added to the ZnO solution and ultrasonicated for 0.5 h. The black solution obtained was then stirred for 24 h at 30 °C. Next, 0.44 g Zn(CH_3_COO)_2_·2H_2_O was added and stirred under a given condition. The final products were collected by filtration, washed by deionized water and ethanol, and finally dried at 60 °C in air for 24 h.

### 2.2. Materials characterization

The structure and morphology of the samples were characterized by X-ray diffraction (XRD, RIGAKU SCXmini), X-ray photoelectron spectroscopy, scanning electron microscope (SEM, JSM-6700F), transmission electron microscope (TEM, Tecnai G2 F20), X-ray photoelectron spectroscopy (XPS, ESCALAB 250) and Raman spectroscopy (Renishaw, excited at 785 nm), respectively.

### 2.3. Electrochemical measurements

The electrochemical behaviors were measured *via* CR2025 coin-type test cells assembled in a dry argon-filled glove box. The test cell consisted of working electrode (∼1.5 mg cm^−2^) and lithium sheet which were separated by a Celgard 2300 membrane and electrolyte of 1 M LiPF_6_ in EC : EMC : DMC (1 : 1 : 1 in volume). The working electrode consisted of 80 wt% active material, 10 wt% carbon black and 10 wt% polymer binder (carboxymethyl cellulose, Na-CMC). The electrodes were dried at 100 °C for 12 h in a vacuum. Cyclic voltammetry tests were operated on a CHI660D Electrochemical Workstation with a scan rate of 0.50 mV s^−1^. The cells were cycled by LAND 2001A at room temperature and different low temperatures. Electrochemical impedance measurements were carried out by applying an ac voltage of 5 mV over the frequency range from 1 mHz to 100 kHz.

## 3. Results and discussion

**Fig. 1:**
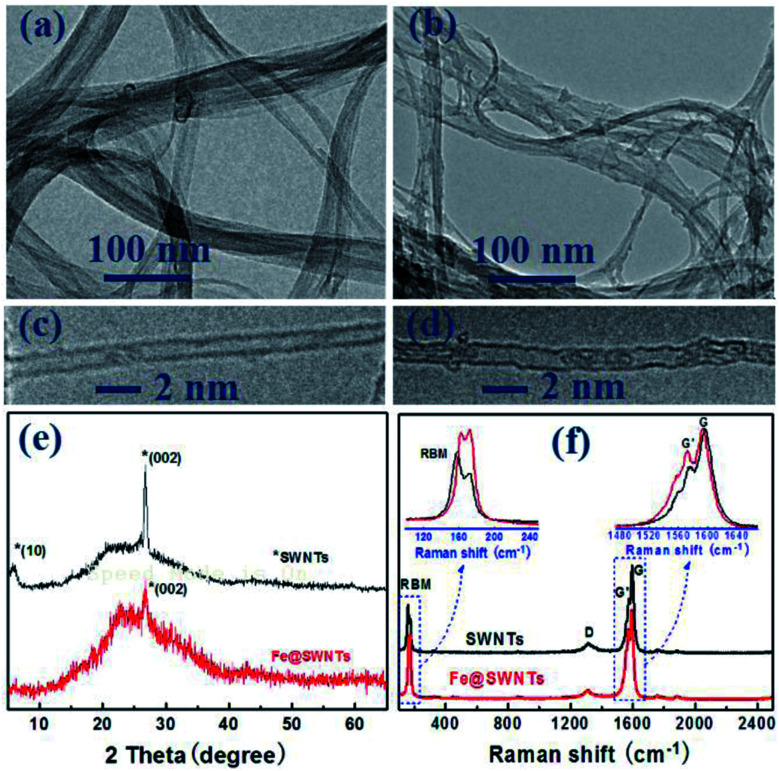
TEM and HR-TEM images of (a and c) SWNTs and (b and d) Fe@SWNTs; (e) XRD patterns of SWNTs and Fe@SWNTs; (f) Raman spectra of SWNTs and Fe@SWNTs.

As observed from Fig. 1(a), the SWNTs appear as 20–80 nm bundles with part appearing as individuals. The Fig. 1(c) reveals that the typical diameter of a SWNT is ∼1.4 nm and the two parallel dark lines correspond to the SWNT walls. After encapsulating with iron particle, Fig. 1(b) displays the same bundle structure for Fe@SWNTs. Compared with Fig. 1(c), Fig. 1(d) clearly shows that segmented dark objects aligned linearly along the tube axis of SWNTs are attributed to iron particles. The other HR-TEM and SEM images for Fe@SWNTs have been shown in Fig. S1 and S2.†Fig. 1(e) compared the XRD patterns of SWNTs and Fe@SWNTs. The peak at 5.8° marked with an asterisk, which was ascribed to hexagonally packed SWNTs, disappeared in Fe@SWNTs because of the contribution of the reduced structure factor of Fe particles inside the tube. The result confirms that the Fe particles filled the tubes of the SWNTs. This result is also supported by the Raman test shown in Fig. 1(f). Fig. 1(f) shows the Raman spectra of the SWNTs and Fe@SWNTs with the excitation wavelength of 785 nm. The insets show the enlarged radial breathing mode (RBM) and G regions. With Fe encapsulation, both RBM and G bands shifted slightly because of the charge transfer between Fe and host SWNTs. The G band of the Fe@SWNTs downshifted from 1597.2 cm^−1^ to1593.2 cm^−1^, and the G′ band became broaden, indicating that the intercalation of Fe molecules into SWNTs leads to n-doping. These results can provide the improved conductivity for SWNT carrier caused by Fe dopant and further enhance their lithium battery performance.

Fig. S3† shows the XPS spectra of Fe@SWNTs and pure SWNTs. From the Fe2p XPS spectra shown in Fig. S3(b),† it is evident that metallic iron (Fe^0^) at 707.5 eV is present in the as-prepared composites. The Fe2p3/2 peaks at 724.2, 719.8 and 710.8 eV are unique peaks for ferric irons, implying that iron has an affinity to bind to the carbon wall in SWNTs.^13,15^ The result is consistent with the C1s and O1s spectra shown in Fig. S4.† As expected, in the comparison of C1s for pure SWNTs and Fe@SWNTs shown in Fig. S4(a) and (c),† a typical sp^2^ C–C peak at 284.5 eV along with a sp^3^ C=C peak at 285.2 eV indicated in both samples are from the carbon layers in SWNTs, and the appearance of peak at 283.5 eV in the Fe@SWNT sample can be attributed to the electron transfer from nano iron to SWNTs. In addition, as revealed in the O1s XPS spectra in Fig. S4(b) and (d),† peaks of the O1s spectra for both Fe@SWNTs and pure SWNTs are similar. Anyhow, combined with the XRD analysis in the manuscript, these results indicate that most iron species exist as metallic Fe.

**Fig. 2:**
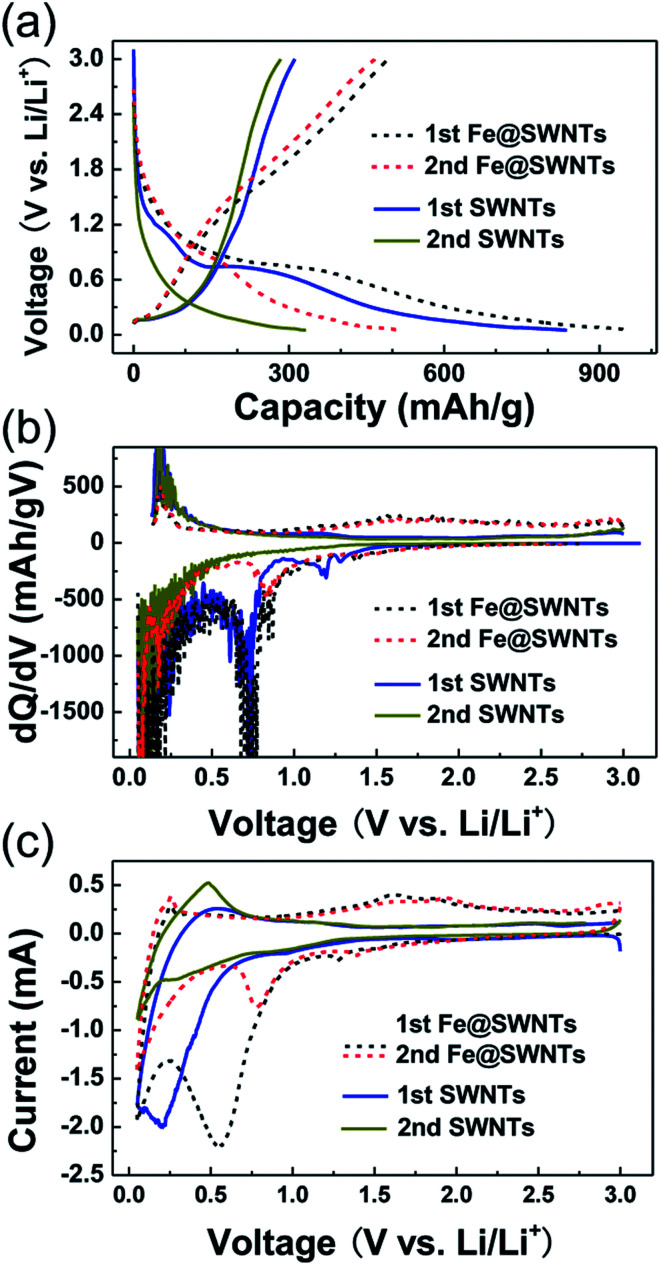
(a) Discharge/charge voltage profiles of the Fe@SWNTs and SWNTs; (b) differential capacity *versus* voltage plots of the Fe@SWNTs and SWNTs corresponding to the first two cycles; (c) cyclic voltammetry curves between 0.05 and 3 V of Li insertion/extraction into/from the Fe@SWNT and SWNT anodes at room temperature. Herein, solid line is marked for pure SWNT anode; dotted line is marked for Fe@SWNT anode.

Electrochemical lithium storage properties of Fe@SWNTs and SWNTs were valuated as anode materials for lithium batteries in half-cell configurations and shown in Fig. 2. From Fig. 2(a), the 1^st^ discharge/charge (D/C) curves of SWNT anode deliver capacities of 302 and 845 mA h g^−1^, indicating a coulombic efficiency of ∼35.6%. In contrast, the first D/C capacities for the Fe@SWNT anode were 505 and 949 mA h g^−1^, with a larger coulombic efficiency of ∼53.1% compared with the SWNTs. Thus, the 1^st^ coulombic efficiency of pure SWNTs can be obviously increased by the encapsulation of catalyst of nano-sized iron, which value reached ∼50% (*i.e.*, ∼50% = (53.1% − 35.6%)/35.6%). In addition to the enhancement of the 1^st^ coulombic efficiency, to confirm the origination of this extra improved reversible capacity is of vital importance.

**Fig. 3:**
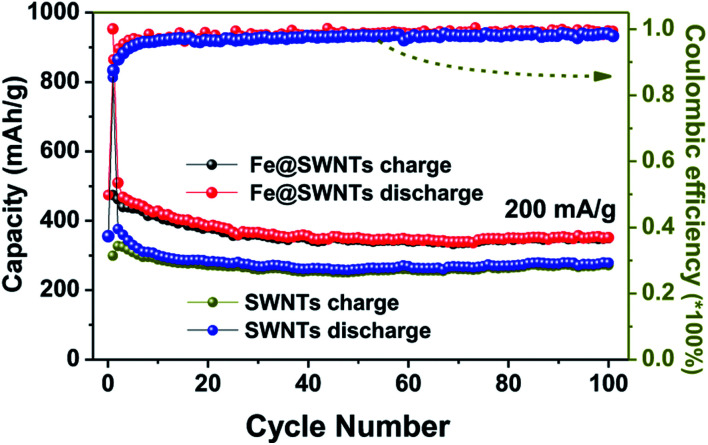
The cycling performance and coulombic efficiency of the Fe@SWNT and SWNT anodes at a current density of 200 mA g^−1^.

In addition, the first two D/C curves of Fe/Fe_3_C–CNFs are quite different from the pure CNFs. To better understand the redox reactions, differential capacity *versus* voltage (d*Q*/d*V*) curves of the first two cycles are investigated in Fig. 2(b). The curves clearly show redox peaks that correspond to the insertion or extraction of Li ions shown in Fig. 2(a). At the first discharge for SWNTs, a broad peak was observed at ∼0.70 V, corresponding to the starting formation of SEI film. This peak greatly decreased at the 2^nd^ cycle, revealing the irreversible formation of SEI components. The following peak from 0.40 to 0.05 V and the extraction peaks around from 0.10 to 0.50 V in the charge were attributed to the Li insertion and extraction in/from trigonal interstitial channels and bundle pores of pure SWNTs, but not their inner channels. The related discussion was displayed in detail in Fig. S5 and S6.† Herein, being similar to SWNTs, the Li cannot insert/extract in/from the inner channels for Fe@SWNTs. However, from the curves of the Fe@SWNTs, the reduction peaks found from 1.30 to 0.80 V in the 1^st^ cycle also appear in the 2^nd^ cycle, implying the formation of reversible SEI films and the additional sites for Li intercalation. In addition, a broad oxidation peak located from 1.20 to 2.20 V being obviously more pronounced in Fe@SWNTs compared with SWNTs, is related to the Li extraction from SEI films and the partial polarization effect. Furthermore, the CVs repeatedly emerged at the same potentials for these two anodes revealed the similar mechanism compared with the d*Q*/d*V* curves. The CVs between 0.05 and 3 V of Fe@SWNTs and SWNTs at room temperature of 25 °C are shown in Fig. 3(c). In addition to the first two CV cycles, the redox peaks at the 3^rd^–8^th^ cycles almost overlap with each other, also indicating the reversible oxidation of some SEI components and good cycling stability (see the additional CVs in Fig. S7†). Based on the above discussion combined with the Raman evidence shown in Fig. S5 and S6,† we attribute the excess Li capacity and distinct redox peaks observed in the Fe@SWNT anodes to Li insert/extract in/from the reversible SEI components. Thus, the nano-sized iron metal encapsulated in SWNTs as catalysts can reduce some SEI components and obviously enhance the lithium battery capacity.

The comparison of the cycling performance of the Fe@SWNTs and SWNTs at a current density of 200 mA g^−1^ was shown in Fig. 3. Apparently, the Fe@SWNTs exhibited a significantly improved reversible capacity. The reversible capacities for these two samples maintain stable with the increasing cycle number up to the 100^th^ cycle. Herein, the reversible capacity of pure SWNTs only remained at ∼275 mA h g^−1^, while that of ∼375 mA h g^−1^ for the Fe@SWNTs, still being similar to the theoretical capacity of commercial graphite of 372 mA h g^−1^. As described in the experimental part, the mass content of Fe in the Fe@SWNTs are ∼17 wt%. Thus, subtracting the contribution from the SWNTs in Fe@SWNT composite, a reversible capacity of ∼863 mA h g^−1^ can be attributed to the additional capacities from SEI film, indicating the good catalytic effect from Fe encapsulation. Herein, the calculated capacity of 863 mA h g^−1^ is based on the following equation: [*C*_Fe@SWNTs_ − (*C*_SWNTs_ × 83 wt%)]/17 wt% = [375 − (275 × 0.83)]/0.17 = 863. Besides to the reversible capacity and the 1^st^ coulombic efficiency discussed at the above part, the coulombic efficiency in the following cycles for Fe@SWNTs of ∼99.3% are larger than of 97.8% for pure SWNTs, which indicating an important lithium battery factor for possible commercial research. The enhanced result is similar to the other recent reports.^26–28^

**Fig. 4:**
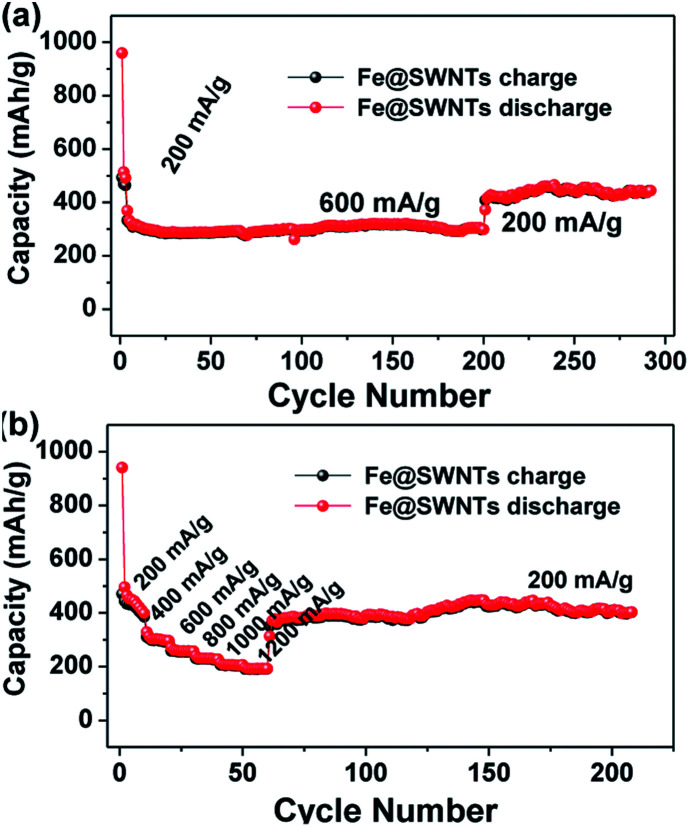
The long cycling performance and the rate cycling performance of the Fe@SWNT anodes at different current densities.

For the Fe@SWNT anodes, the further test of the long cycling and rate performance under different current densities have been carried out and displayed in Fig. 4. From Fig. 4(a), the cycle performance tested at relatively high current densities of 600 mA g^−1^ after being activated at 200 mA g^−1^ in the first three cycles. This anode can deliver reversible capacities of ∼300 mA h g^−1^ tested at 200 mA g^−1^ and 395 mA h g^−1^ tested at 600 mA g^−1^ for 197 and 95 cycles, respectively. Moreover, each fairly stable capacity at different current densities from 200 to 1200 mA g^−1^ can be observed in Fig. 4(b). When the current was reduced back to 200 mA g^−1^, the Fe@SWNTs can still deliver a reversible capacity of ∼390 mA h g^−1^, implying the good rate stability. These results indicate a good long cycling and rate performance. Furthermore, the long cycling and rate performance for pure SWNTs under the same tested condition of Fe@SWNTs have been carried out and compared in Fig. S8 and S9.† The results still revealed that the SWNTs with Fe encapsulation can provide better LIB performance including the coulombic efficiency especially for the 1^st^ cycle, cycling stability, reversible capacity and rate cycling properties.

**Fig. 5:**
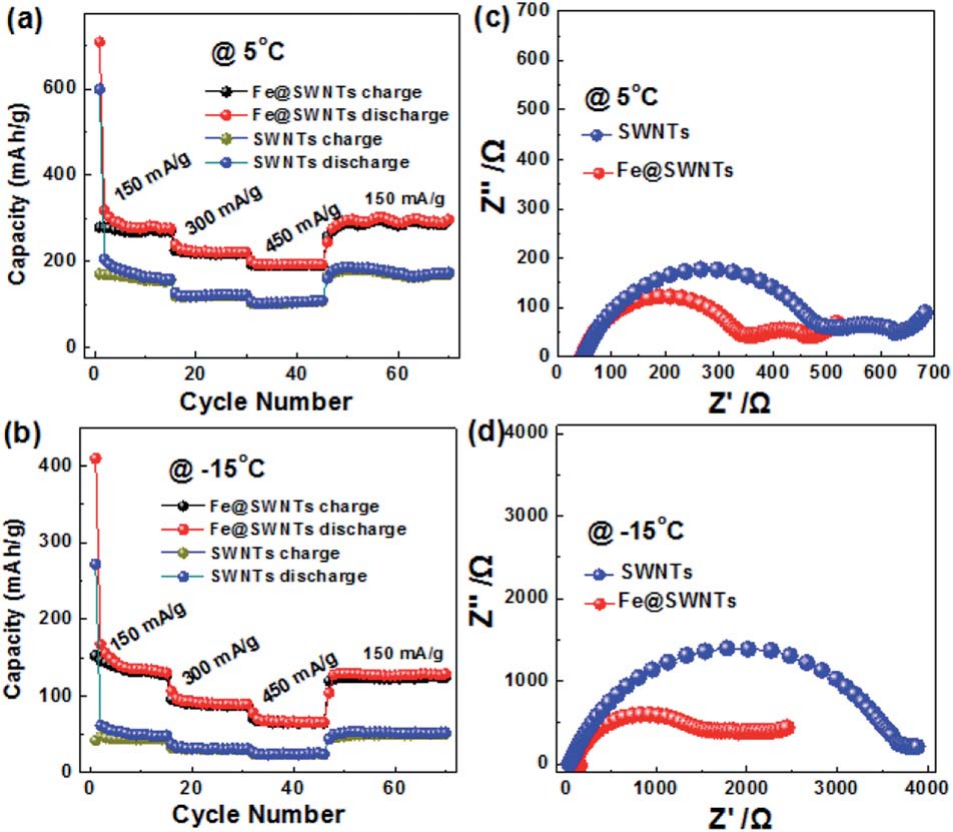
The cycling performance and the corresponding electrochemical impedance spectra of the SWNT and Fe@SWNT anodes: (a and c) 5 °C and (b and d) −15 °C.

As we know, the charge-transfer resistance for metal materials would decrease obviously with decreasing the temperature.^7,13,29^ Furthermore, to prove whether the improved effect of Fe@SWNTs on their LIB performance can remain in low temperature is also of vital importance. Thus, it is interesting and desired to investigate the low-temperature LIB performance for the Fe@SWNT anodes. Fig. 5(a) and (c) show the LIB performance of the SWNT and Fe@SWNT anodes under 5 and −15 °C at different current densities. Each fairly stable capacity tested from 150 to 450 mA g^−1^ can be observed. When the current was reduced back to 150 mA g^−1^, the Fe@SWNTs can still deliver reversible capacities of ∼283 and 142 mA h g^−1^ under 5 and −15 °C, being better than ∼172 and 47 mA h g^−1^ for pure SWNT anodes respectively. Therefore, with the decrease of tested temperature, the Fe@SWNTs shows a capacity loss of ∼49% (*i.e.*, (142 − 283)/283 = 49%). By contrast, the SWNTs show a much larger capacity loss of ∼73%. For further evaluating the enhanced LIB performance for SWNTs with Fe encapsulation, the electrochemical impedance measurements were carried out at 5 and −15 °C after test and shown in Fig. 5(b) and (d). From Fig. 5(b), the difference of total resistance at 5 °C between SWNT and Fe@SWNT anodes are several hundreds of ohm. It is further to interestingly find that this difference increased sharply to several thousands of ohm when the temperature reduced to −15 °C as revealed in Fig. 5(d). As expected, the SWNTs demonstrate a more deteriorated resistance tested at lower temperature without Fe encapsulation. These results indicated that the presence of Fe encapsulation can significantly improve the conductivity and charge transfer resistance for Fe@SWNTs especially at a low temperature, and further enhance their LIB performance.

**Fig. 6:**
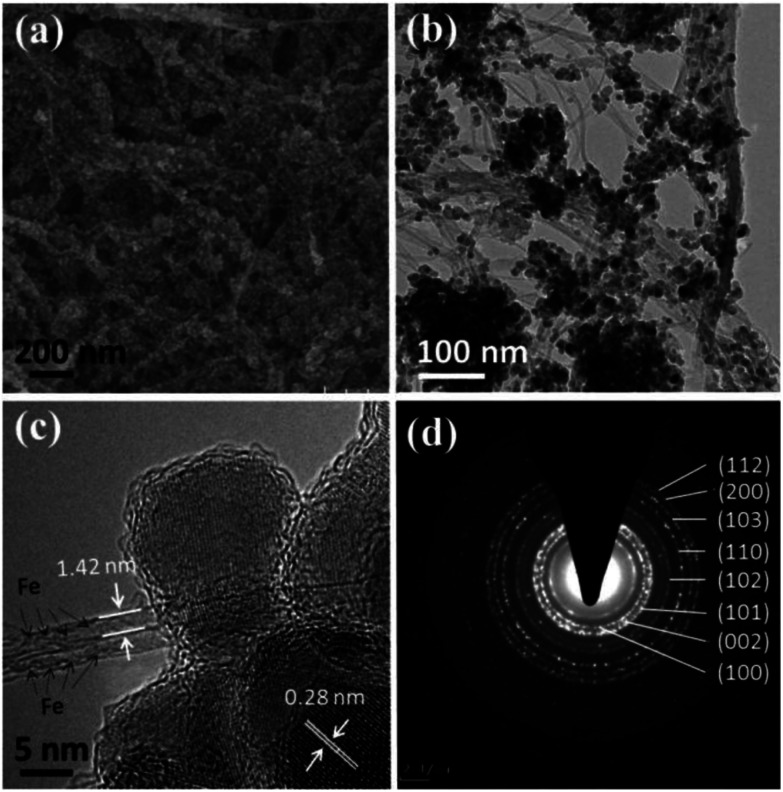
(a) SEM, (b) TEM, (c) HR-TEM images and (d) SAED pattern of ZnO/Fe@SWNTs.

To investigate the enhanced effect of Fe@SWNT carriers for anodes, we preliminarily synthesis the composite of ZnO/Fe@SWNTs and study their LIB performance. The morphology and structure have been characterized by SEM, TEM and SAED, as shown in Fig. 6. As can be seen from Fig. 6(a), the ZnO NPs were coated well on the surface of Fe@SWNTs. The TEM image of Fig. 6(b) also supports this result. The covering of uniform ZnO NPs and typical bundles of Fe@SWNTs can be clearly observed. Fig. 6(c) further shows a HR-TEM image of ZnO/Fe@SWNTs. Being similar to the Fig. 1(d), the typical diameter of the SWNTs can be clearly found to be ∼1.4 nm, and the encapsulated iron filler can also be clearly determined. The existence of iron element was also supported by the EDS result shown in Fig. S9.† Meanwhile, two adjacent planes of ∼0.28 nm corresponding to the interlayer spacing of the (100) plane of ZnO was shown in figure. The samples of ZnO/SWNTs and ZnO/Fe@SWNTs were characterised and compared by XRD in Fig. S10.† The peak at 26° is ascribed to the (002) graphitic plane in SWNTs. All of other eight diffraction peaks could be unambiguously assigned to the zincite (ZnO) (JCPDS card no. 36-1451).^30,31^ These results are in consonance with that SEAD analysis. The detailed diffraction rings were presented in Fig. 6(d). Eight diffraction rings, respectively corresponding to the (103), (110), (102), (101), (002), (100), (200) and (112) planes of ZnO, can be observed clearly. In order to confirm the loading ratios of ZnO, SWNTs and Fe@SWNTs in composites, these two composite was used for TG analysis as shown in Fig. S11.† From the TGA curves, the weight ratio of the ZnO in ZnO/SWNTs and ZnO/Fe@SWNTs are ∼58.4 wt% and ∼56.1 wt%, respectively.

**Fig. 7:**
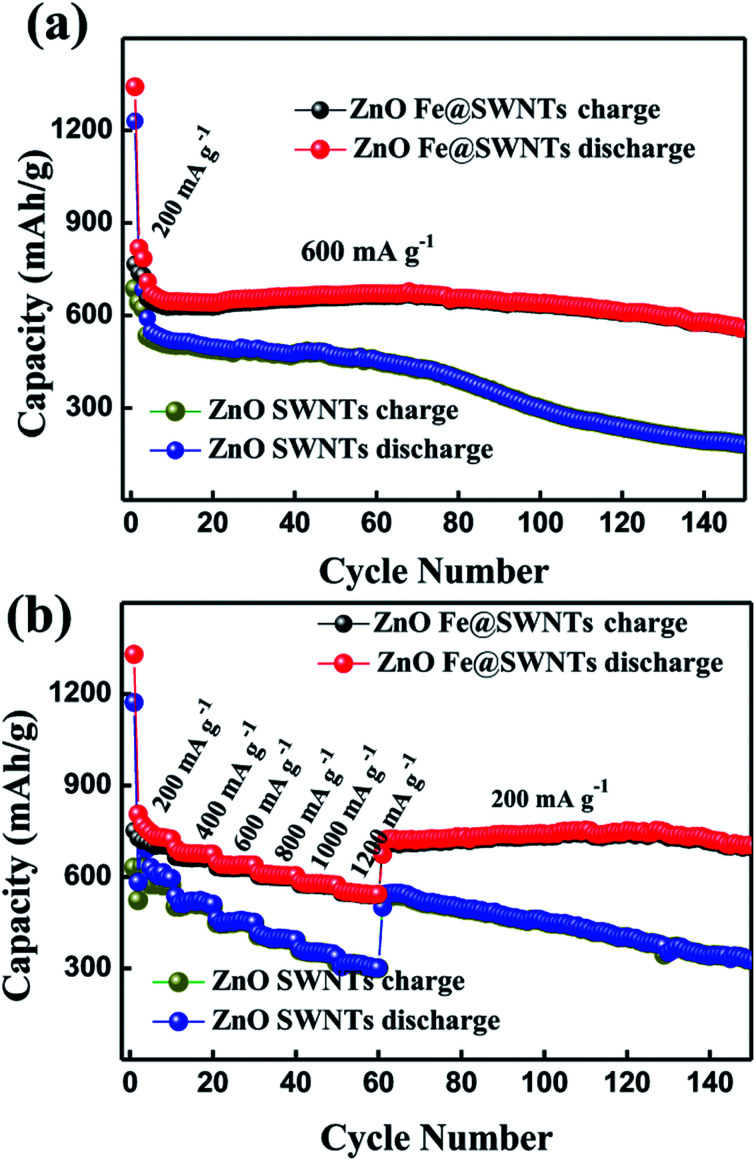
The cycling performance and the rate cycling performance of the ZnO/SWNT and ZnO/Fe@SWNT anodes at different current densities.

The Fig. 7 compared the cycling performance and the rate cycling performance of the ZnO/SWNT and ZnO/Fe@SWNT anodes at different current densities. Herein, Fig. S12a and b† show the discharge/charge voltage profiles of the ZnO/SWNT and ZnO/Fe@SWNT anodes. Equating to the CVs, the differential capacity *versus* voltage (d*Q*/d*V*) curves of the first two cycles are investigated in Fig. S12c and d.† The related electrochemical redox mechanism has been discovered for Fig. S12c and d, as shown in ESI.† As displayed in Fig. S13,† the two samples were tested at current densities of 200 mA g^−1^. Apparently, the ZnO/Fe@SWNTs exhibited a significantly improved reversible capacity. Meanwhile, these two samples were also tested at current densities of 600 mA g^−1^ after activating at 200 mA g^−1^ in the first three cycles as shown in Fig. 7(a). As expected, the ZnO/Fe@SWNT anode demonstrates an obviously better LIB cycling stability than that of without Fe encapsulation. The anode of ZnO/Fe@SWNTs can deliver discharged capacity of ∼575 mA h g^−1^ after 150 cycles at 600 mA g^−1^, showing a relative stable cyclability. However, the discharge capacity fade presented after the 60^th^ cycle, remaining at only ∼195 mA h g^−1^ after 150 cycles. From Fig. 7(b), when the tested current densities increased from 200 mA g^−1^ to 1200 mA g^−1^, the initial reversible capacity of ∼720 mA h g^−1^ for ZnO/Fe@SWNT anode decreased to ∼545 mA h g^−1^, showing a difference of 175 mA h g^−1^. Under the same condition, the reversible capacity for ZnO/Fe@SWNT anode decreased from ∼583 to ∼316 mA h g^−1^, delivering a larger capacity fade of 267 mA h g^−1^ compared to ZnO/Fe@SWNT anode. More importantly, as the current density returned to 200 mA g^−1^, the ZnO/Fe@SWNT anode delivered a high capacity of ∼725 mA h g^−1^ even after 150 cycles, being much better than that of ∼335 mA h g^−1^ for the ZnO/SWNT anode. Interestingly, as calculated in the TGA result, the ZnO/SWNTs and ZnO/Fe@SWNTs contain ∼58.5 and ∼56 wt% ZnO, respectively. Thus, based on the Fig. 3 and S13,† subtracting the contribution from Fe@SWNTs in ZnO/Fe@SWNT nanocomposite, a reversible capacity of 958 mA h g^−1^ can be attributed to ZnO, indicating 98% of the theoretical capacity of ZnO (978 mA h g^−1^). By measuring at 200 mA g^−1^, the capacity of 958 mA h g^−1^ and the ratio of 98% can be calculated by the following equations: [*C*_ZnO/Fe@SWNTs_ − *C*_Fe@SWNTs_ × (44.0 wt%)]/56.0 wt% = [700 − (375 × 0.44)]/0.56 = 958 and 958/978 = 98%. The reversible capacity of ZnO in ZnO/Fe@SWNT nanocomposite can almost achieve its theoretical capacity under low current densities in this current study. The lithium battery performance presented in this work is comparable to the related reports.^30–33^ It is concluded that the electrochemical performance of in ZnO/Fe@SWNT nanocomposite composite can be strongly improved by using Fe@SWNTs as supporting carriers and catalysts.

Furthermore, from Fig. S14,† the cycle performance tested at relatively low temperature of 5 °C and at relatively high current densities of 1000 mA g^−1^ after being activated at 200 mA g^−1^ in the first three cycles. This anode can deliver reversible capacities of ∼330 mA h g^−1^ tested at 1000 mA g^−1^ even for 1400 cycles, revealing the excellent long cycling performance at low temperature. It is also concluded that the presence of Fe encapsulation can significantly improve the conductivity and charge transfer resistance for ZnO/Fe@SWNTs, being beneficial for bearing varied cycling currents. These results revealed that the ZnO/Fe@SWNT anode with good conductivity during the cycling process, which conclusion was also proved by the EIS result as shown in Fig. S15.†

## 4. Conclusions

In summary, to obtain the improved coulombic efficiency has become the key topic for LIB anodes of nano-carbon materials. As a typical sample, the novel composites of Fe-encapsulated single-walled carbon nanotubes (Fe@SWNTs) were synthesized *via* a simple vapor phase method coupled with heat treatment. Then the nano-structure Fe@SWNTs can be obtained and used as anode materials for LIBs. The resulting Fe@SWNT anode can provide much larger coulombic efficiency of 53.1% in the 1^st^ cycle than 35.6% for pure SWNTs, implying the value increment reached ∼50%. Meanwhile, compared with pure SWNT anodes, the Fe@SWNTs can exhibit much better LIB performance both at room temperature and low temperatures of 5 and −15 °C. Importantly, the origination of this extra improved reversible capacity has been confirmed to be derived from the reversible reaction of SEI film activated by the catalyst. This preliminary work provides a useful strategy to obtain improved coulombic efficiency and reversible capacity nano-carbon anodes for high performance LIBs. Moreover, the preliminary result revealed that Fe@SWNTs used as high performance carriers for attaching ZnO NPs can deliver improved LIB performance.

## Conflicts of interest

There are no conflicts to declare.

## Supplementary Material

RA-008-C8RA00480C-s001
