# Supplementary material for: Iron encapsulated in single-walled carbon nanotubes for obtaining the evidence of improved coulombic efficiency and improving the lithium battery performance of ZnO anodes
Source: RSC Adv. 2018 Mar 23;8(21):11566–73. doi: 10.1039/c8ra00480c (PMC9079148; doi:10.1039/c8ra00480c)
Supplement: RA-008-C8RA00480C-s001 [file RA-008-C8RA00480C-s001.pdf]

Electronic Supplementary Material (ESI) for RSC Advances.
This journal is © The Royal Society of Chemistry 2018

Electronic Supplementary Material (ESI) for

Iron encapsulated in single-walled carbon nanotubes for obtaining the evidence of improved coulombic efficiency and improving the lithium battery performance of ZnO anode

Jiaxin Li,[abc] Mingzhong Zou,[bc] Weijian Huang,[bc] Chuxin Wu,[a] Yi Zhao,[a] Lunhui Guan,[a]* Zhigao Huang[bc]*

a CAS Key Laboratory of Design and Assembly of Functional Nanostructures and Fujian Provincial key Laboratory of Nanomaterials, Fujian Institute of Research on the Structure of Matter, Chinese Academy of Sciences, Fuzhou, Fujian 350002, PR China.

b College of Physics and Energy, Fujian Normal University, Fujian Provincial Key Laboratory of Quantum Manipulation and New Energy Materials, Fuzhou， 350117, China.

c Fujian Provincial Collaborative Innovation Center for Optoelectronic Semiconductors and Efficient Devices, Xiamen, 361005, China

*Telephone/Fax: 86-591-22867577. E-mail: guanlh@fjirsm.ac.cn, zghuang@fjnu.edu.cn

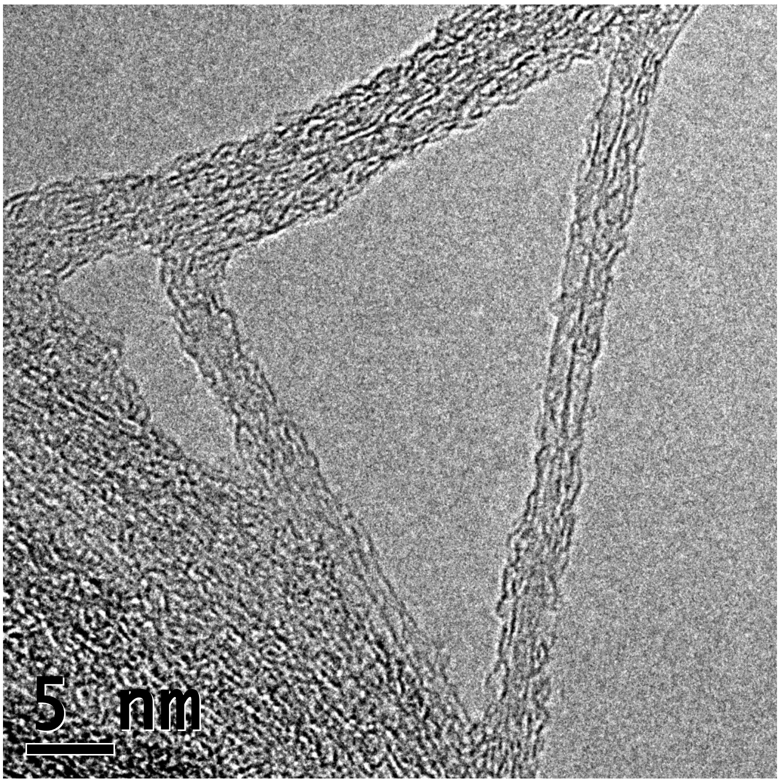

Fig. S1. HR-TEM image of Fe@SWNTs.

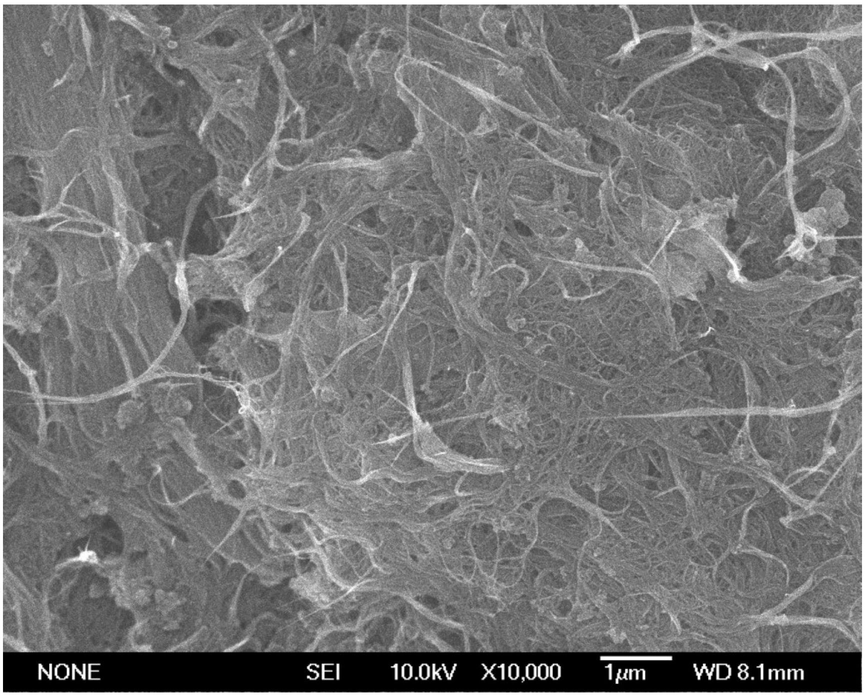

Fig. S2. SEM image of Fe@SWNTs.

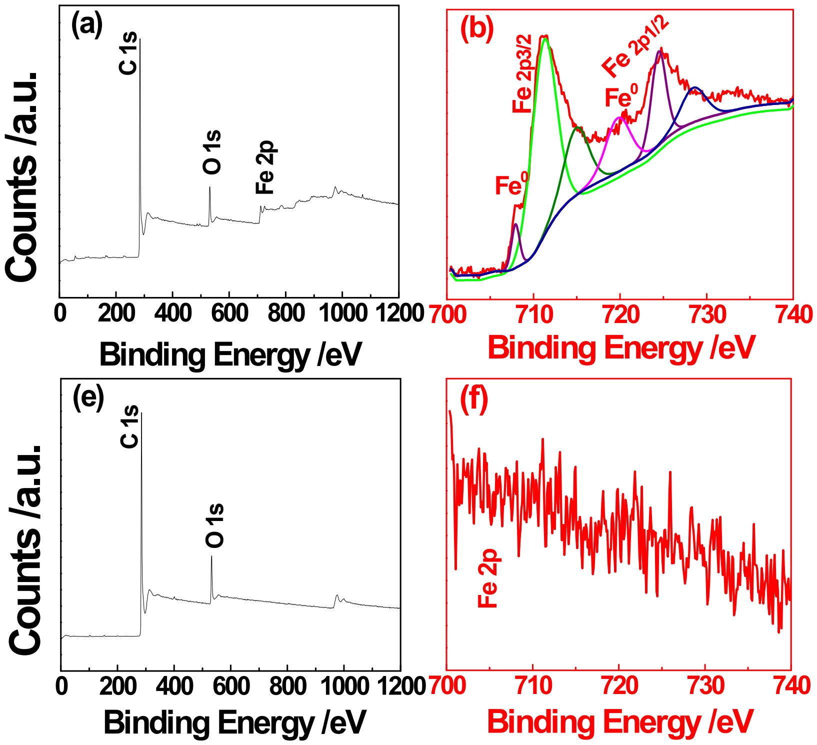

Fig. S3. XPS spectra for (a,b) Fe@SWNTs and (c,d) SWNTs.

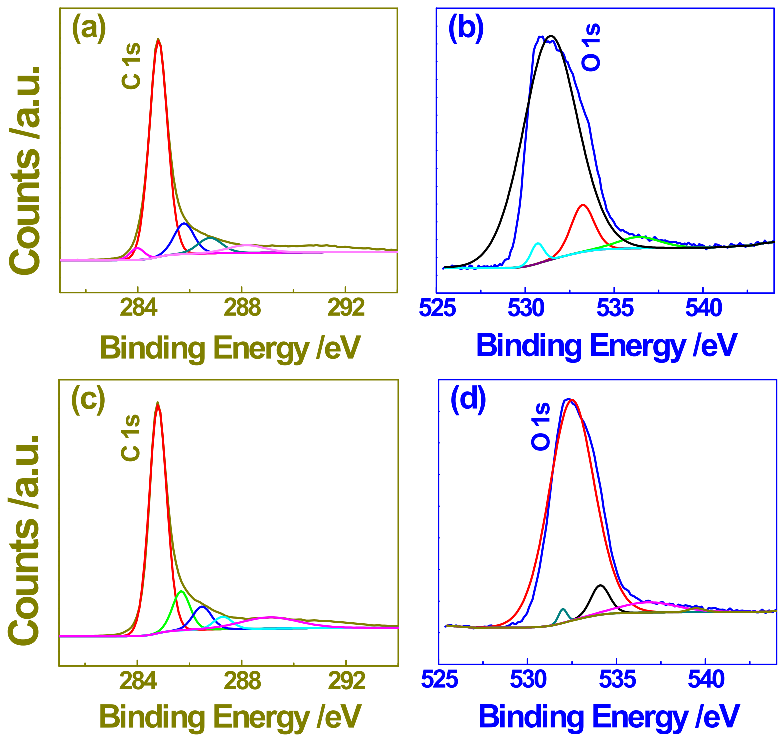

Fig. S4. XPS spectra for (a,b) Fe@SWNTs and (c,d) SWNTs.

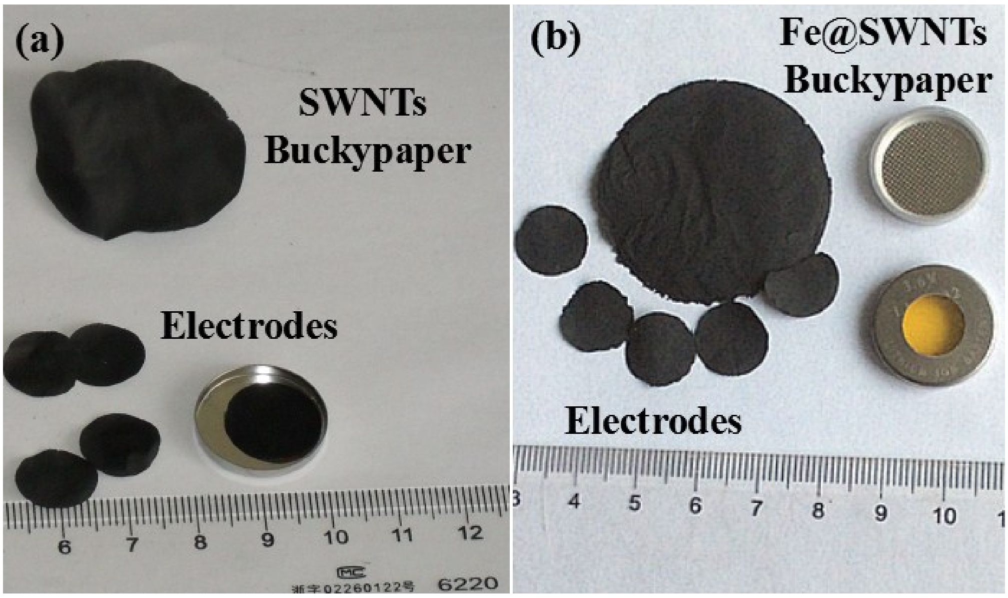

Fig. S5. The photograph of different (a) SWNTs and (b)Fe@SWNTs buckypapers(BP) and BP-electrodes.

About 16-18 mg of SWNT and Fe@SWNT materials were respectively dispersed in 100 mL deionized H_2O under the assistance of ultrasonic for a long time. For each sample, the suspension was filtered with a 0.45 um microfilm. The microfilm adhered with SWNT or Fe@SWNT related materials were dried under IR lamp. After drying, the black deposit on the film was self-peeled into a BP disk. Then the BP was cut into a 1.25 cm disk of about 1.8~2.0 mg for each electrode, which was denoted as BP electrode. The BP electrodes were measured via opening CR2025 coin-type test cells with membrane protection assembled in a dry argon-filled glove box. The cells were cycled by LAND 2001A at room temperature with different discharge/ charge conditions, and further used to carry out the Raman measurements.

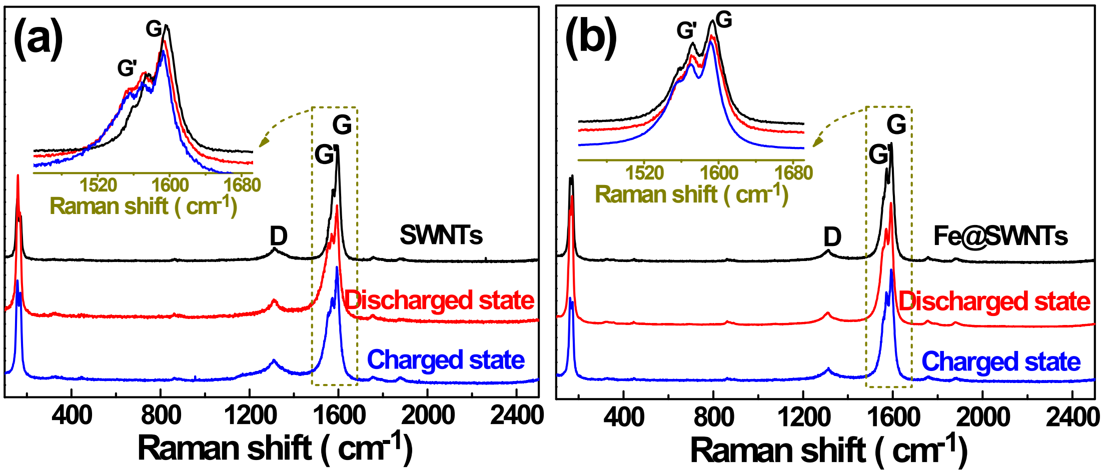

Fig. S6. Raman spectra for (a) SWNTs and (b) Fe@SWNTs electrodes during different discharge/charge progresses.

The following is the discussion of Li Storage in SWNT and Fe@SWNT anodes [1-5]. To increase our understanding about the Li storage behavior and the charge-transfer effect between the SWNTs (or Fe@SWNTs) and Li ions, Raman detection was carried out. Fig. S5 presents the Raman spectra of SWNT-BP and Fe@SWNT-BP electrodes at discharged to 0.05V and recharged to 3.00V, respectively. Based on the previous reports, the G peak is quite sensitive to doping: p-doping induces an upshift, while n-doping induces a downshift. For pure SWNTs, the shifts for G peak were probably derived from the competition between the upshift of the G-band caused by the stress induced by the insertion of the Li ions in interstitials of tubes and the downshift caused by the effect of the charge transfer from the Li ions inserted inside the tubes. Furthermore, as shown in the manuscript, the G peak for Fe@SWNTs downshifted by ~4cm^{-1} compared with the pristine SWNTs, resulting from electron transfer from the iron to the host SWNTs. This result is similar to the K@SWNTs in our previous work [6]. As observed from Fig. S5(a), the G-band

downshifted to 1594.1cm^{-1} after discharged to 0.05 V, and upshifted to 1592.8 cm^{-1}after recharged to 3.00 V. This result revealed that the Li ions can enter the inner space of the tubes, the trigonal interstitial channels of the bundles, and the intercalation sites between adjacent tubes. The dominant reversible sites for lithium storage for SWNTs were the trigonal interstitial channels and and the intercalation sites between adjacent tubes. Being similar to the SWNTs, the G-band for Fe@SWNTs (shown in Fig. S5(b)) downshifted from 1593.2 to 1592.1cm^{-1} after discharged to 0.05 V, and further downshifted to 1590.8 cm^{-1}after recharged to 3.00 V, revealing that the Li ion entered the inner space of the tubes cannot reversible extract from these sites.

Here, the Li ion storage mechanism in SWNTs and Fe@SWNTs has been investigated clearly. Thus, SWNTs, as a good confined nano-vessel, can be used to fill nano iron particles and further to enhance their catalystic activity for LIBs. Besides, this strategy can effectively provide evidence for the additional reversible capacity produced from the reversible reaction of SEI film.

Refs.:

[1] J.-X. Li, Y. Zhao, L.-H. Guan, Electrochemistry Communications, 12 (2010) 592.

[2] J. Li, C. Wu, L. Guan, The Journal of Physical Chemistry C, 113 (2009) 18431.

[3] Kawasaki, S., Iwai, Y., Hirose, M. Mater. Res. Bull. 2009, 41, 415.

[4] Kim, Y. A., Kojima, M., Muramatsu, H., Umemoto, S., Watanabe, T., Yoshida, K., Sato, K.,; Ikeda, T., Hayashi, T., Endo, M., Terrones, M., Dresselhaus, M. S. Small 2006, 2, 667.

[5] Iwaia, Y., Hirosea, M., Kanoa, R., Kawasakia, S., Hattorib, Y., Takahashic, K. J. Phys. Chem. Solids 2008, 69, 1199.

[6] N. Wang, L. Guan, Nanoscale, 2 (2010) 893.

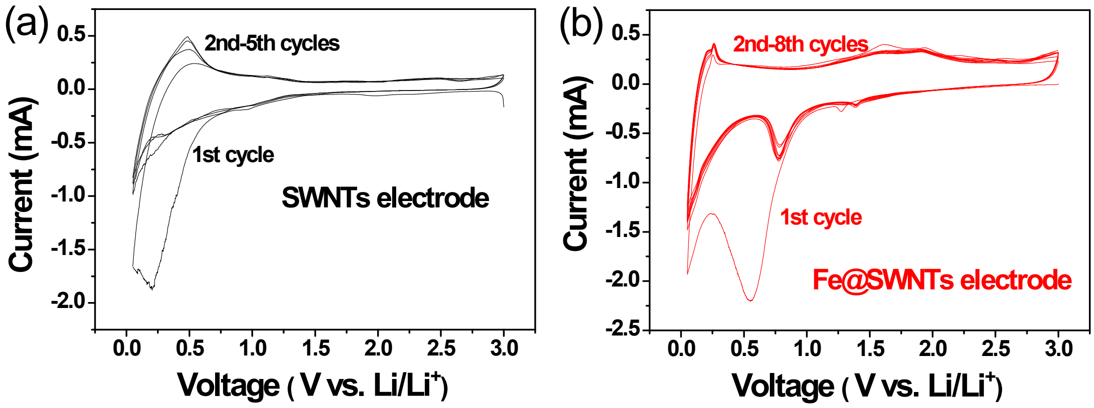

Fig. S7. Cyclic voltammetry curves between 0.05 and 3 V of Li insertion/extraction into/from the Fe@SWNT and SWNT anodes at room temperature.

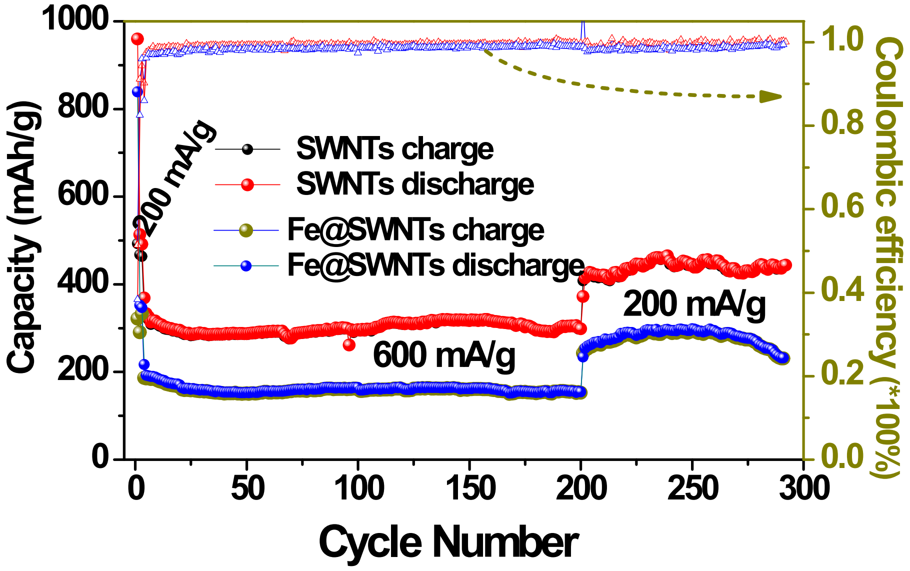

Fig. S8. The long cycling performance and coulonbic efficiency of the Fe@SWNT and SWNT anodes at current densities of 200 and 600 mA/g.

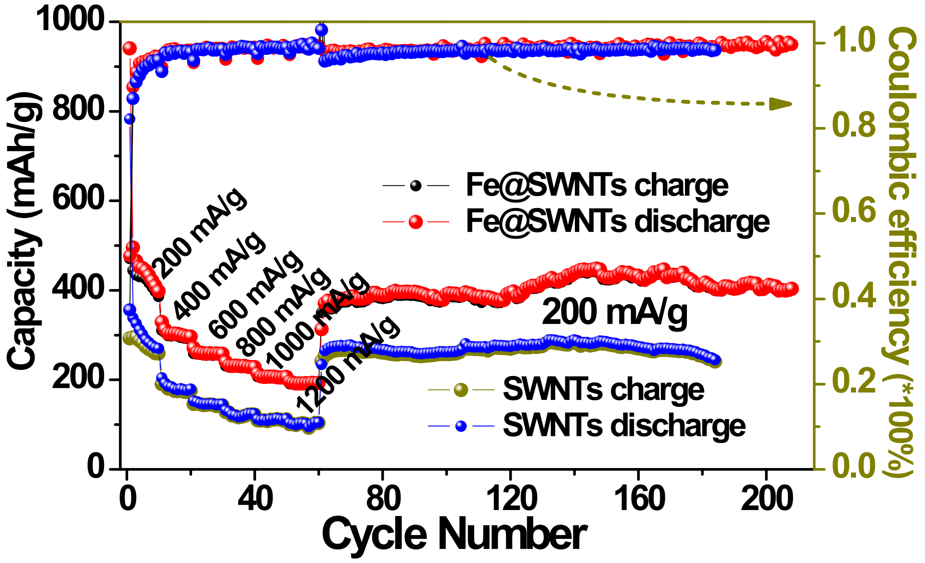

Fig. S9. The rate cycling performance and coulonbic efficiency of the Fe@SWNT anodes at different current densities.

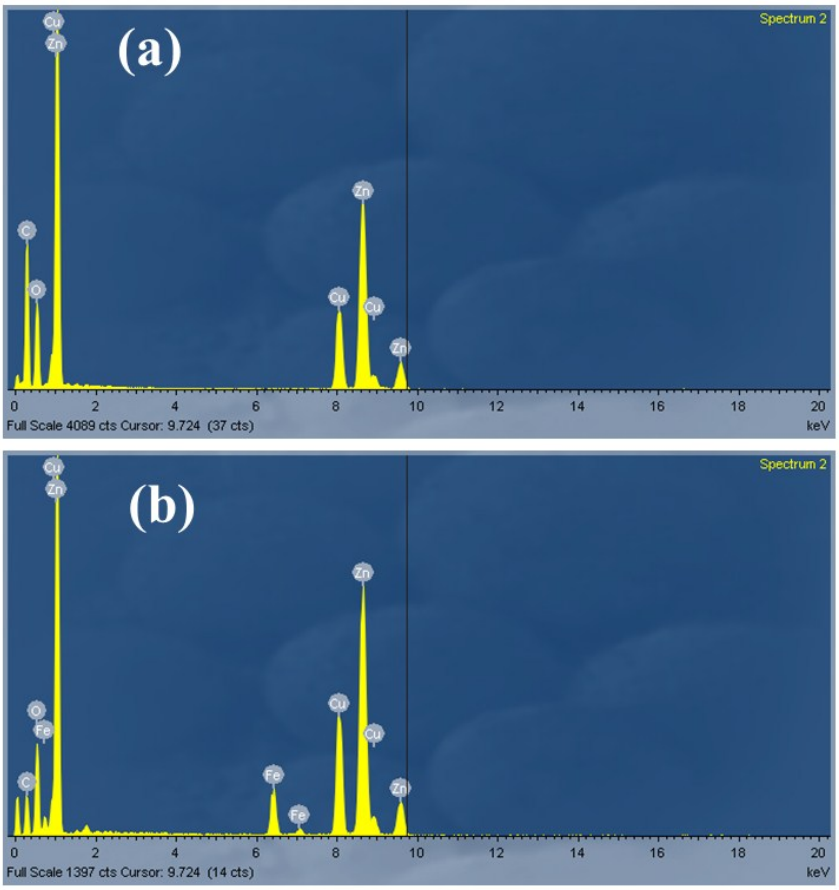

Fig. S10. The EDS patterns of (a) ZnO/SWNTs and (b) ZnO/Fe@SWNTs.

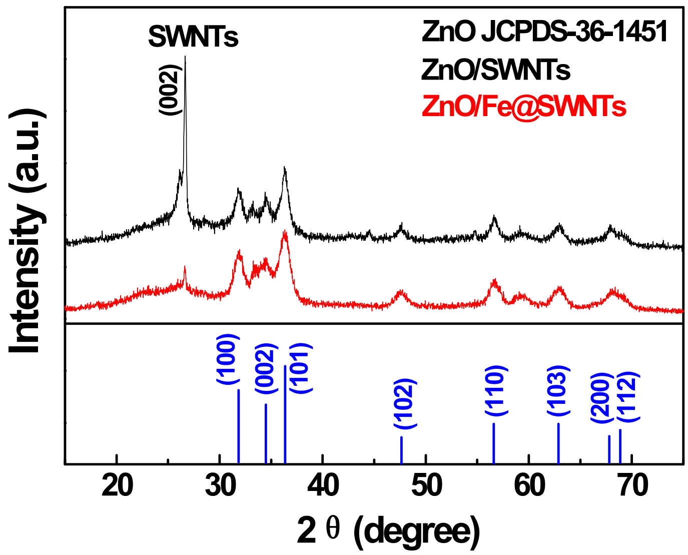

Fig. S11. The XRD patterns of (a) ZnO/SWNTs and (b) ZnO/Fe@SWNTs.

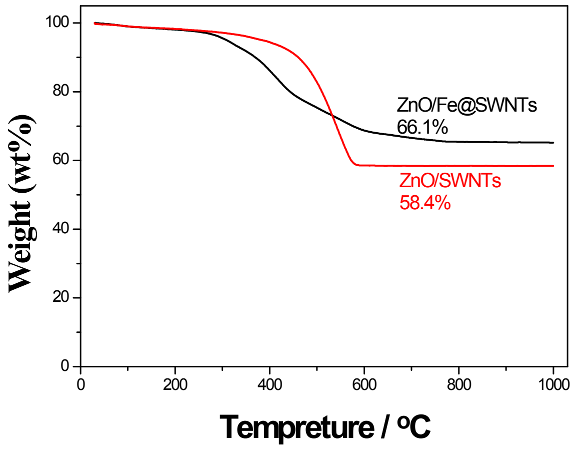

Fig. S12. TGA curve of ZnO/SWNT and ZnO/Fe@SWNT nanocomposite.

In order to confirm the loading ratios of ZnO, SWNTs and Fe@SWNTs in composites, these two composite was used for TG analysis as shown in **Fig. S12**. From the TGA for ZnO@SWNTs, the weight ratio of the ZnO, SWNTs in ZnO/SWNTs nanocomposite are ~58.4 wt.% and ~42.6 wt.%, respectively. For ZnO/Fe@SWNTs, the sample is heated to 1000 °C in air so that Fe is oxidized to Fe_2O_3 and carbon is oxidized to CO_2. Thus, the weight of Fe_2O_3 products from Fe transformation is 1.428 times than that of Fe (i.e., divided by their molecular weight, 160 / 112 = 1.428). Based on the weight of remaining ZnO and the Fe_2O_3 product, the calculated equation can be displayed as the following: ZnO wt.% = 100 wt.% - SWNT wt.% - Fe_2O_3 wt.% = 100 wt.% - 33.9 wt.%- (33.9 wt.% * (17 wt.% / 83 wt.%) * 1.428) = ~56.1 wt.%. Herein, the weight ratio of the Fe in Fe@SWNT sample is ~17%.

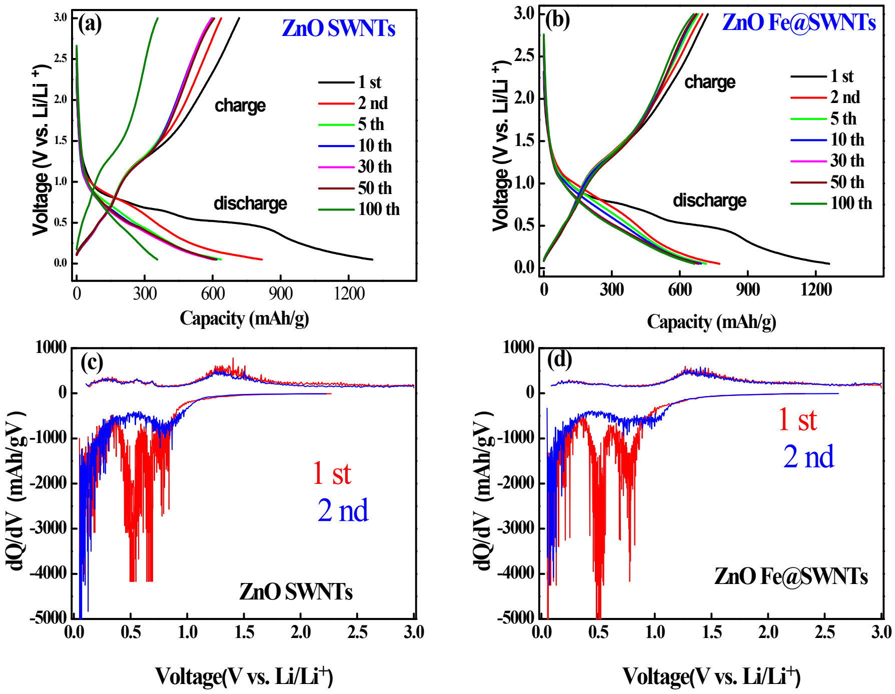

Fig. S13. (a,b) Discharge/charge voltage profiles of the ZnO/SWNT and ZnO/Fe@SWNT anodes, respectively; (c,d) differential capacity versus voltage plots of the ZnO/SWNT and ZnO/Fe@SWNT anodes of the first two cycles.

Figs. S13a and **b** show the discharge/charge voltage profiles of the ZnO/SWNT and ZnO/Fe@SWNT anodes. Equating to the CVs, the differential capacity versus voltage (dQ/dV) curves of the first two cycles are investigated in figs 3c and d. From figs 3c and d, the strong peaks between 0.30~0.85 V in the first cathodic scan are related to the first electrochemical process of the anode materials. This process contains the reduction of ZnO into Zn, the formation of Li-Zn alloy, and the growth of the solid electrolyte interphase (SEI) layer. And the peaks extending to 0.05V correspond to the insertion of lithium ions into SWNTs. In the first anodic scan, the peaks located from 0.25 to 0.70V are ascribed to the multi-step dealloying process of Li-Zn alloy. The strong peaks at 1.58 V correspond to the convention of Zn to ZnO. After the 1st cycle, the cathodic peaks locate at 0.50 and 0.85 V, relating with the alloying processes. The redox peaks of both of ZnO/SWNT and ZnO/Fe@SWNT anodes in the 2nd cycle overlap well with those in the 1st cycle, indicating their better electrochemical reversibility and structural stability.

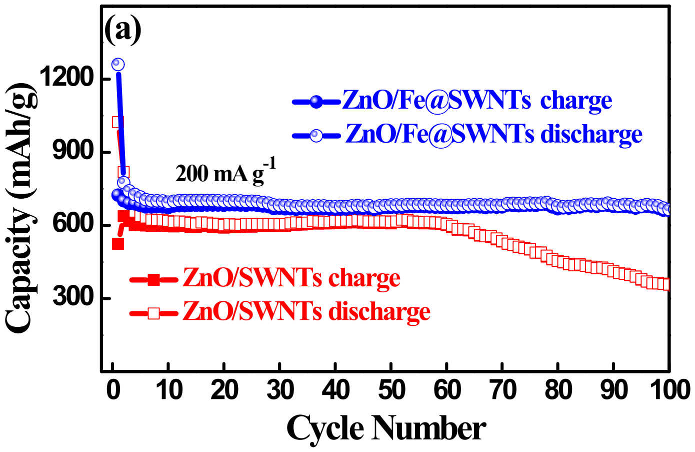

Fig. S14. The cycling performance of the ZnO/SWNT and ZnO/Fe@SWNT anodes at 200 mA g^{-1}.

Fig. S14 compared the cycling performance of ZnO/SWNT and ZnO/Fe@SWNT anodes at a current density of 200 mA g^{-1}. Apparently, the ZnO/Fe@SWNTs exhibited a significantly improved reversible capacity. The reversible capacities for these two samples maintain stable with the increasing cycle number up to the 55th cycle. However, the reversible capacity of ZnO/SWNT anode decreased and only remained at ~350 mAh g^{-1} after at the 100th cycle. As expected, the ZnO/Fe@SWNT anode demonstrates an obviously better LIB cycling stability than that of without Fe encapsulation.

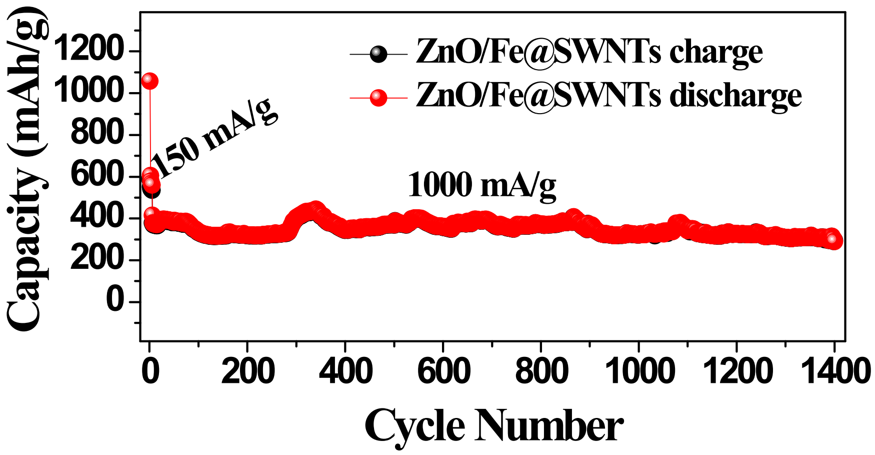

Fig. S15. The cycling performance of the ZnO/Fe@SWNT anode at relatively low temperature of 5 °C.

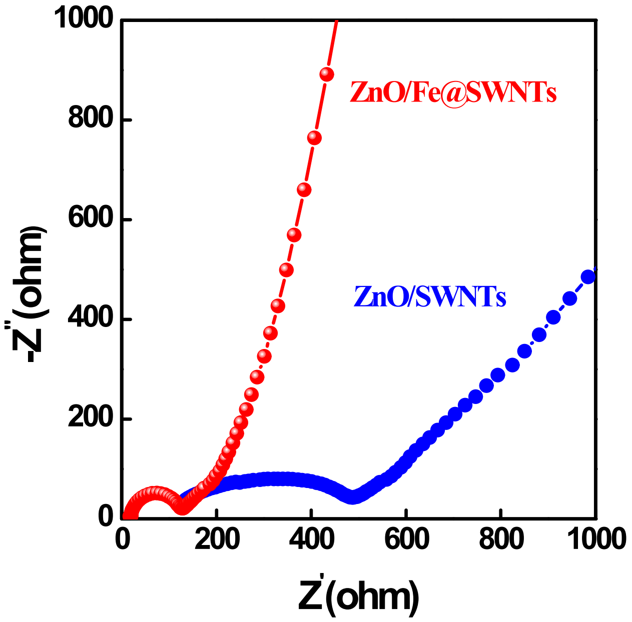

Fig. S16. Impedance analysis of ZnO/SWNT and ZnO/Fe@SWNT anodes after long tested at room temperature.
